# Efficacy of mHealth Interventions for Improving Maternal and Neonatal Outcomes Among Pregnant Women With Hypertensive Disorders: Protocol for a Systematic Review

**DOI:** 10.2196/51792

**Published:** 2023-11-28

**Authors:** Judith Angelitta Noronha, Mitchelle S Lewis, Tenzin Phagdol, Baby S Nayak, Anupama D, Jyothi Shetty, Ravishankar N, Sreekumaran Nair

**Affiliations:** 1 Department of Obstetrics and Gynecological Nursing Manipal College of Nursing Manipal Academy of Higher Education Udupi India; 2 Department of Fundamentals of Nursing Manipal College of Nursing Manipal Academy of Higher Education Manipal India; 3 Department of Child Health Nursing Manipal College of Nursing Manipal Academy of Higher Education Manipal India; 4 Department of Global Health Prasanna School of Public Health Manipal Academy of Higher Education Manipal India; 5 Department of Obstetrics and Gynecology Kasturba Medical College Manipal Academy of Higher Education Manipal India; 6 Department of Biostatistics Vallabhai Patel Chest Institute University of Delhi New Delhi India; 7 Medical Biometrics & Informatics (Biostatistics) JIPMER Pondicherry India

**Keywords:** digital health, gestational hypertension, hypertension, hypertensive, knowledge synthesis, maternal health outcomes, maternal, mHealth, mobile health application, mobile health, neonatal health outcomes, neonatal, neonates, preeclampsia, pregnancy, pregnant, review methodology, review methods, SMS, systematic, telemedicine, text messaging, sustainable development goal

## Abstract

**Background:**

Hypertension is one of the most prevalent medical conditions that arise during pregnancy, resulting in maternal and neonatal complications. Mobile health (mHealth) has emerged as an innovative intervention for delivering maternal and child health care services. The evidence on the effectiveness of mHealth interventions in improving the health outcomes of pregnant women with hypertensive disorders is lacking. Therefore, there is a need for evidence synthesis using systematic review methods to address this evidence gap.

**Objective:**

This review aims to determine the efficacy of mHealth interventions in improving maternal and neonatal outcomes among pregnant women with hypertensive disorders. The review will answer the following research questions: (1) What are the types of mHealth interventions used in pregnant women with hypertensive disorders? (2) Are the various mHealth interventions effective in improving maternal and neonatal health outcomes, health behaviors, and their knowledge of the disease? and (3) Are mHealth interventions effective in supporting health care providers to make health care decisions for pregnant women with hypertensive disorders?

**Methods:**

This review will include randomized controlled trials, nonrandomized controlled trials, and cohort studies focusing on mHealth interventions for pregnant women with hypertensive disorders. Studies reporting health care providers use of mHealth interventions in caring for pregnant women with hypertensive disorders will be included. The search strategy will be tailored to each database using database-specific search terms. The search will be conducted in PubMed-MEDLINE, ProQuest, CINAHL, Scopus, Web of Science, and CENTRAL. Other literature sources, such as trial registries and bibliographies of relevant studies, will be additionally searched. Studies published in English from January 2000 to January 2023 will be included. A total of 2 review authors will independently perform the data extraction and the quality appraisal. For quality appraisal of randomized controlled trials, the Cochrane Risk of Bias 2 tool will be used. The Risk of Bias in Nonrandomized Studies of Interventions (ROBINS-1) tool will be used for nonrandomized controlled trials, and the Critical Appraisal Skills Programme checklist for cohort studies will be used. Any disagreements between the 2 reviewers will be resolved through discussion and a third reviewer if required. A meta-analysis will be performed based on the availability of the data.

**Results:**

As per the protocol, the study methodology was followed, and 2 independent reviewers conducted the search in 6 databases and clinical registries. Currently, the review is in the full-text screening stage. The review will publish the results in the first quarter of 2024.

**Conclusions:**

The evidence synthesized from this systematic review will help guide future research, support health care decisions, and inform policy makers on the effectiveness of mHealth interventions in improving the maternal and neonatal outcomes of pregnant women with hypertensive disorders.

**International Registered Report Identifier (IRRID):**

PRR1-10.2196/51792

## Introduction

### Background

Hypertensive disorders of pregnancy (HDP) complicate 5% to 10% of pregnancies, leading to maternal, fetal, and neonatal morbidity and mortality [1]. According to American College of Obstetricians and Gynecologists guidelines, hypertension in pregnancy is classified as chronic or preexisting hypertension, gestational hypertension, preeclampsia or eclampsia, chronic or preexisting hypertension with superimposed preeclampsia-eclampsia, mild hypertension, and severe hypertension [2]. Globally, the incidence of hypertensive disorders during pregnancy had a 10.92% increment from 16.30 million (95% uncertainty interval [UI] 13.56-19.42 million) to 18.08 million (95% UI 15.26-21.11 million) in the last decade from 1990 to 2019. Regionally, the highest incidence of HDP in 2019 was detected in South Asia (3.84 million, 95% UI 3.16-4.62 million), followed by western sub-Saharan Africa (3.71 million, 95% UI 2.64-3.63 million) and eastern sub-Saharan Africa (3.12 million, 95% UI 2.64-3.63 million), respectively. Conversely, Australasia (30,110, 95% UI 23,080-38,670), Oceania (37,060, 95% UI 29,640-46,290), and Central Europe (78,060, 95% UI 64,040-94,230) had the lowest incidence estimates. There were approximately 27,830 deaths due to hypertensive disorders during pregnancy in 2019. Countries with low sociodemographic and human development indexes have an increasing burden [3]. According to the World Health Organization (WHO), maternal mortality remains “unacceptably high” worldwide every day, with some 830 women dying from a preventable cause of pregnancy or childbirth-related complications [4]. Most of these deaths (94%) that occurred in low-resource settings could have been prevented. High blood pressure during pregnancy is a major complication that accounts for nearly 75% of maternal deaths [5]. Therefore, tackling hypertension during pregnancy is an important global public health concern to date.

Some of the significant risk factors for HDP include a higher BMI, anemic conditions, and poor education. Maternal age, primiparity, multiple pregnancies, HDP in a previous pregnancy, gestational diabetes mellitus, preexisting hypertension, preexisting type 2 diabetes mellitus, preexisting urinary tract infection, a family history of hypertension, type 2 diabetes mellitus, and preeclampsia are possible nonmodifiable risk factors [6]. HDP was also associated with poor maternal, perinatal, and neonatal outcomes, especially in pregnant women with preeclampsia and eclampsia [7]. A higher burden of stillbirth was found in multiparous women with HDP [8]. A systematic review and meta-analysis reported that pregnant women with chronic hypertension showed high pooled incidences of superimposed preeclampsia (25.9%), cesarean section (41.4%), preterm delivery (28.1%), low birth weight (16.9%), neonatal unit admission (20.5%), and perinatal death (4%) [9]. Therefore, the identification and management of HDP should take priority in pregnant women to avoid preventable deaths and morbidities among mothers and their babies.

There is a moderate quality of evidence that recommends antihypertensive therapy for the management of HDP [10]. Single interventions are not likely to significantly reduce pregnancy-related mortality and morbidity. Instead, improving the overall quality of antenatal care across the health care system is required [11].

An innovative health intervention increasingly being used and tested in maternal and child health is mobile health (mHealth). mHealth refers to “the use of wireless and mobile information and communication technologies to support health and health care” [12]. Globally, mobile phone use is continuously expanding [13]. The International Telecommunication Union reported that about 4.9 billion people were using the internet in 2021. This has increased by 17% since 2019 [14]. The portability, instantaneous access, and direct communication capabilities of mobile technology enable prompt dissemination of health information, which improves medical and public health practices cost-effectively [15]. These qualities characterize mHealth, which enables better health care access and facilitates improved delivery of health care services. In the face of this technological reality, mHealth plays an innovative role in the delivery of health care. However, though the global use of mHealth is increasing, the effect it has on the improvement of outcomes is still unknown [16]. Although current evidence suggests that telemonitoring provides benefits for managing patients at high risk for HDP, more research is needed to prove its safety and effectiveness [17].

### Aim

This review aims to collate relevant data and provide evidence related to the effectiveness of mHealth interventions in improving maternal and neonatal outcomes among pregnant women with hypertensive disorders. This review will also identify the evidence gap in mHealth for antenatal care, which will guide further research, support health care professionals in their practice decisions, and inform policy makers as well as other stakeholders to implement changes in the health care system.

## Methods

### Overview

The systematic review protocol was designed based on the PRISMA-P (Preferred Reporting Items for Systematic Review and Meta-Analysis Protocols) 2015 checklist [18]. The review has been registered in PROSPERO (CRD42022321686). The review will be conducted in accordance with the *Cochrane Handbook for Systematic Review of Interventions* [19].

### Study Eligibility Criteria

The inclusion and exclusion criteria for the review are given in Textbox 1.

**The inclusion and exclusion criteria.**

**Inclusion criteria**

- Randomized controlled trials, nonrandomized controlled trials, and cohort studies
- Studies with the population of pregnant women with hypertensive disorders (chronic hypertension; gestational hypertension; preeclampsia-eclampsia; chronic hypertension with superimposed preeclampsia; and the “hemolysis, elevated liver enzymes, low platelet count” syndrome) and health care providers using mobile health (mHealth) in providing care for pregnant women with hypertensive disorders
- mHealth interventions that use mobile technology, such as mobile phones, personal digital assistants, and other wireless devices such as wearables and tracking sensors, to support the health of pregnant women with hypertensive disorders and health worker decisions
- Studies with a control group receiving standard care, no intervention, other forms of digital interventions, antenatal counseling, dietary plans, yoga, and any form of breathing exercises
- Maternal mortality, maternal morbidities, perinatal death, and neonatal morbidities; knowledge of the disease and its management; and health behaviors such as early identification of warning signs
- English language
- Conducted between January 2000 and January 2023
- All settings across the globe

**Exclusion criteria**

- Noninterventional observational studies
- Other high-risk pregnancies, such as gestational diabetes; pregnant women with autoimmune diseases, fibroids, and thyroid disorders; and postnatal women with hypertensive disorders
- Nonmobile digital devices such as desktop computers, which are used for health management information systems and electronic health records
- Studies without comparison groups
- Outcomes other than health outcomes, such as the usability or feasibility of mHealth interventions
- Languages other than English

### Search Methods for Identification of Studies

A search will be conducted in the following databases: PubMed-MEDLINE, ProQuest, CINAHL, Scopus, Web of Science, and CENTRAL by 2 independent reviewers (TP and AD). Medical Subject Headings (MeSH) terms, subject headings, and keywords will be searched electronically in all fields. Search strings will be developed using database-specific filters and tailored to each database using the search given in Multimedia Appendix 1.

### Searching Other Sources

Additionally, ClinicalTrials.gov, the Clinical Trial Registry of India, and the WHO International Clinical Trials Registry platform will be searched for unpublished and ongoing trial reports. The reference list of retrieved articles will also be searched.

### Data Management

#### Selection of Studies

The first stage of the screening process will involve 2 authors who will independently screen the titles and abstracts of the included studies (TP and AD). They will independently scrutinize the studies for inclusion and exclusion criteria with a prespecified form. For studies with insufficient data in the abstract, the full text will be retrieved and assessed for eligibility. If there is any disagreement between the 2 authors, they will discuss it, and if needed, the third author (ML) will be asked for an opinion. If any inexplicit data are found or any data are missing, then the authors of the corresponding study will be contacted.

#### Data Extraction

Data will be extracted independently by 2 authors (TP and AD) using a standard data extraction form developed by the review authors (BN and JS). The variables recorded will be the authors’ name, publication year, study period, journal of publication, the country where the study was performed, participant age, sample size, variables adjusted for in the analysis, and findings. The data extracted from each included study will be based on the following: type of mHealth interventions used, outcomes assessed, funding source, type of publication, source of the study if not published, and other study details necessary for risk of bias assessment. Disagreements will be resolved by discussion with the third author (JN).

#### Quality Appraisal

A total of 2 review authors (TP and AD) will independently assess the risk of bias in studies. The authors will independently assess randomization sequence generation, treatment allocation concealment, blinding, completeness of outcome data, selective outcome reporting, and other sources of bias for randomized controlled trials using the Cochrane Risk of Bias 2 tool [19]. The Risk of Bias in Nonrandomized Studies of Interventions (ROBINS-1) tool will be used to appraise the quality of nonrandomized controlled trials [20]. The Critical Appraisal Skills Programme checklist for cohort studies will be used [21]. We will resolve disagreements, if any, by discussing them with the third author to reach a consensus.

#### Data Synthesis and Analysis

Statistical analysis will be performed by SN and RN, referencing the *Cochrane Handbook for the Systematic Review of Intervention*. If there are adequate data, a meta-analysis will be performed using a random-effects model. Categorical data will be summarized using the odds ratio, relative risk, or hazard ratio. Continuous data will be summarized using the standardized mean difference. A forest plot will be generated. Sensitivity analysis will be performed if applicable.

#### Assessment of Heterogeneity and Bias Reporting

A funnel plot and Egger test will be performed to assess publication bias. When data from included studies can be pooled, statistical heterogeneity will be assessed by visual inspection of forest plots using the chi-square test and the *I*^2^ statistic.

#### Subgroup Analyses

Trial characteristics (participants, design, interventions, outcomes, and risk of bias) will be examined, and based on them, subgroup analysis will be performed.

However, if we are not able to perform a meta-analysis due to insufficient studies or heterogeneity, the findings will be reported descriptively.

### Ethical Considerations

The systematic review will be done using previously available data and will not include any personal information about individual participants. As a result, permission from a research ethics committee is not required.

## Results

As per the protocol, the study was conducted following the methodology according to the *Cochrane Handbook for Systematic Review of Interventions*. A total of 2 independent reviewers conducted the search in the following 6 databases: PubMed-MEDLINE, ProQuest, CINAHL, Scopus, Web of Science, and CENTRAL. Additional sources, such as clinical trial registries (ClinicalTrials.gov, the Clinical Trial Registry of India, and the WHO International Clinical Trials Registry), were searched using the search strategy developed by the team. Currently, the review is in the full-text screening stage, and data collection is ongoing. The findings of the review will be published in the first quarter of 2024.

## Discussion

### Overview

This review is designed to evaluate the effectiveness of mHealth interventions on maternal and neonatal health outcomes among pregnant mothers with hypertensive disorders.

mHealth interventions are implemented and researched extensively. Evidence from systematic reviews shows that mHealth interventions are effective in increasing the use of antenatal and postnatal care, including attendance at antenatal and postnatal care check-ups, facility-based deliveries, and skilled attendance at birth and vaccination rates [22]. Conversely, another systematic review and meta-analysis concluded that most studies conducted in low- and middle-income countries are of poor methodological quality, and few have evaluated impacts on patient outcomes [23]. Therefore, there is a need for a rigorous systematic review that addresses health end points as primary or secondary outcomes and includes recent studies and rigorous study designs that are considered the gold standard components to determine an intervention’s effect, that is, a randomized controlled trial. Apart from health outcomes, evidence also indicates that health workers can be empowered to accept and use mHealth in circumstances where it is appropriate for their requirements, workload, training, and abilities [24]. In turn, mHealth may provide health care professionals with skills and confidence when technology is viewed as beneficial and simple to use in settings that encourage acknowledgment from clients, peers, or superiors.

The review will enable the critical appraisal and synthesis of evidence on the use of mHealth interventions among pregnant women with hypertensive disorders. As there is a rapid increase in technological development and the relative newness of mobile apps as a tool for health care providers in the management of hypertensive disorders during pregnancy, more primary research has been published [25]. Therefore, the evidence pointing toward the use of these services must be put together, which will help in formulating local policies that can be generalized and promulgated for use in all health care settings and communities. Additionally, the extent to which clear conclusions can be drawn about the usefulness of mHealth interventions in terms of health outcomes may be limited. However, this review will provide clarity on the existing evidence and its implications for health care providers.

### Limitations

The possible limitations might be the inclusion of nonrandomized controlled trials and cohort studies that may not provide a strong evidence base. However, we planned to include those in this review as we do not intend to miss any available evidence. The strength of the evidence depends on the quality of the involved studies. The publication bias of the included studies might affect the results, but they will be reported. Meta-analysis and meta-regression may not be possible due to different methodological approaches in varying studies; however, subgroup analysis will be done.

### Conclusion

The review results will highlight the available evidence on mHealth apps and their effectiveness in terms of maternal and neonatal health. The review results provide directions for further practice and further research in this area. It will inform policy makers and those in the hospital and community settings to implement mHealth interventions at the local level as well as scale up to a wider population.

## Appendix

Multimedia Appendix 1 Search strategy (PICOS).

## Abbreviations

HDP: hypertensive disorders of pregnancy
MeSH: Medical Subject Headings
mHealth: mobile health
PRISMA-P: Preferred Reporting Items for Systematic Review and Meta-Analysis Protocols
ROBINS-1: Risk of Bias in Nonrandomized Studies of Interventions
UI: uncertainty interval
WHO: World Health Organization

## Data Availability

Data sharing is not applicable to this article as no data sets were generated or analyzed in this study protocol.
