# Supplementary figures and images for: Efficacy of mHealth Interventions for Improving Maternal and Neonatal Outcomes Among Pregnant Women With Hypertensive Disorders: Protocol for a Systematic Review
Source: JMIR Res Protoc. 2023 Nov 28;12:e51792. doi: 10.2196/51792 (PMC10716747; doi:10.2196/51792)

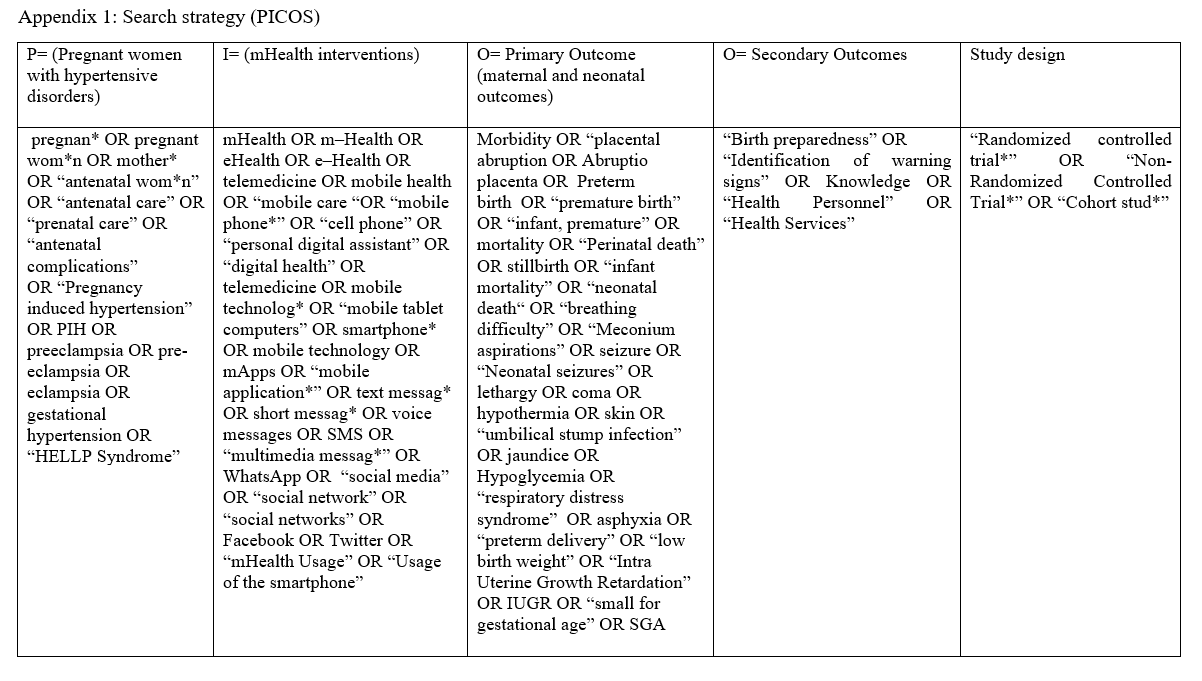

Supplement: Multimedia Appendix 1 [file resprot_v12i1e51792_app1.png]
